# Cation permeability in CorA family of proteins

**DOI:** 10.1038/s41598-020-57869-z

**Published:** 2020-01-21

**Authors:** Artem Stetsenko, Albert Guskov

**Affiliations:** 1 Groningen Biomolecular & Biotechnology Institute, University of Groningen, Nijenborgh 7, 9747 AG Groningen, the Netherlands; 2 Moscow Institute of Physics and Technology, Dolgoprudny, Russia

**Keywords:** Ion channels, Membrane proteins

## Abstract

CorA proteins belong to 2-TM-GxN family of membrane proteins, and play a major role in Mg^2+^ transport in prokaryotes and eukaryotic mitochondria. The selection of substrate is believed to occur via the signature motif GxN, however there is no consensus how strict this selection within the family. To answer this question, we employed fluorescence-based transport assays on three different family members, namely CorA from bacterium *Thermotoga maritima*, CorA from the archeon *Methanocaldococcus jannaschii* and ZntB from bacterium *Escherichia coli*, reconstituted into proteoliposomes. Our results show that all three proteins readily transport Mg^2+^, Co^2+^, Ni^2+^ and Zn^2+^, but not Al^3+^. Despite the similarity in cation specificity, ZntB differs from the CorA proteins, as in the former transport is stimulated by a proton gradient, but in the latter by the membrane potential, confirming the hypothesis that CorA and ZntB proteins diverged to different transport mechanisms within the same protein scaffold.

## Introduction

**Figure 1:**
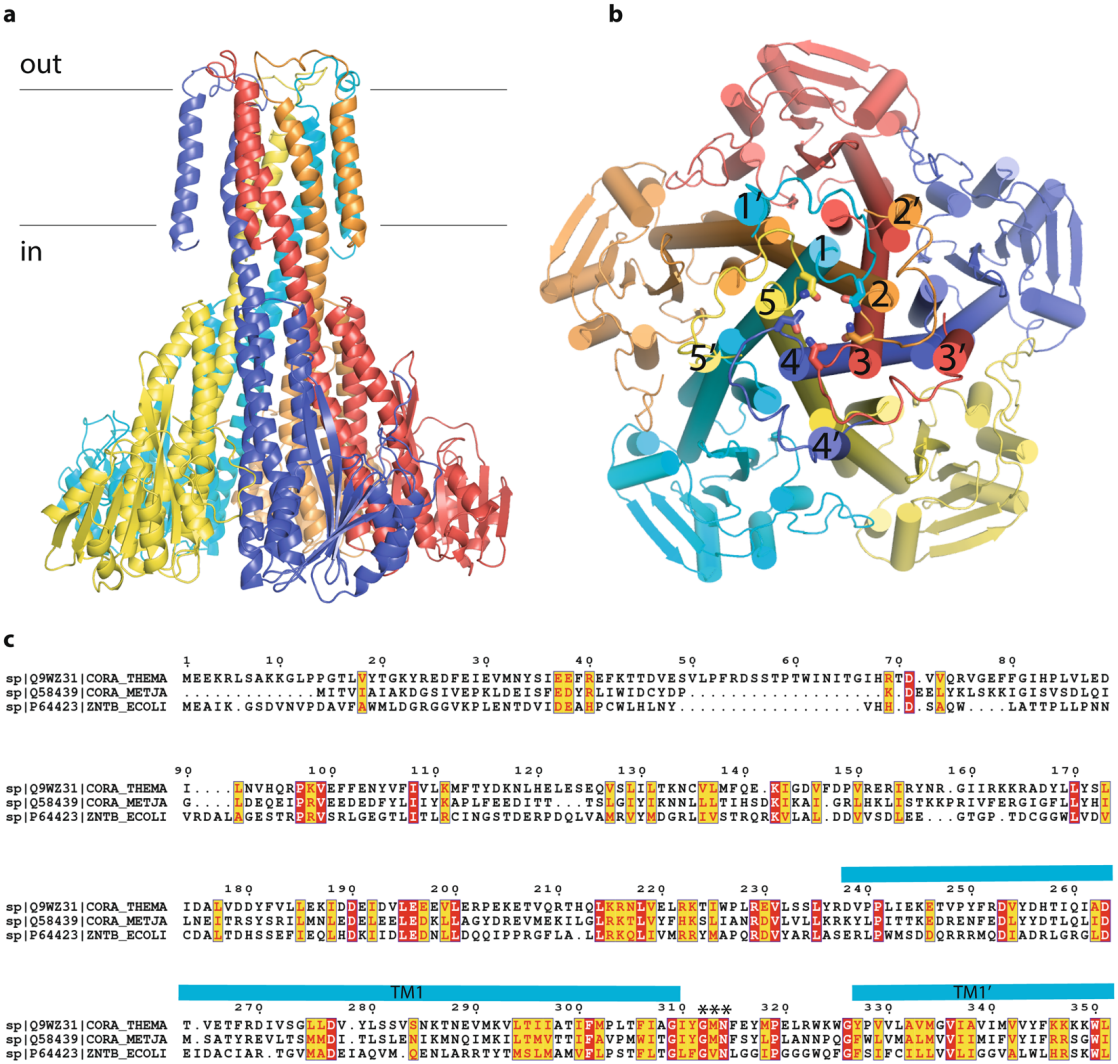
The general organization of CorA family of proteins as exemplified by TmCorA (pdb code 4I0U). (**a**) side view, each protomer is color-coded; the position of membrane is indicated with black lines; (**b**) view from the extracellular part, transmembrane helices are numbered with the numerals, those with ′ indicate the outer transmembrane helix of each protomer. The asparagine side chains of GxN motif are shown as sticks; (**c**) the sequence alignment of TmCorA, MjCorA and EcZntB. Essentially conserved amino acids are in red. The turquoise bars show the position of the long helices (numbered in panel b) forming the channel and of the periphery helices (indicated with ′ in panel b). The signature motif/selectivity filter is indicated with *. The sequence alignment was produced with T-coffee^42^ (http://tcoffee.crg.cat/apps/tcoffee/index.html) and annotated with Espript 3.0 (ref. ^43^) (http://espript.ibcp.fr).

Magnesium is one of the essential metal ions, which is invariantly required for every cell, as it is involved in numerous metabolic reactions and also plays additional roles, for example as a stabilizer of highly charged adenosine triphosphate and lipidic bilayer, where it compensates the negative charge of phosphate groups^1^. Since magnesium is normally present in biological systems in its ionic form as Mg^2+^, it cannot readily cross the biological membrane, thus it is channeled via membrane-embedded proteins^2^. In prokaryotes, this is often done via MgtE and CorA families of proteins^2,3^. The latter are homo- or hetero- pentamers^2,4–7^, possess large cytoplasmic domains, which are believed to play a regulatory function^8,9^ and the transmembrane part, which consists of two α-helices per protomer, arranged as an inner and an outer pentamer, while the loops connecting them bear the signature motif GxN, which is at the same time is the selectivity filter^6,10,11^ (Fig. 1).

Most of the efforts in the structural characterization on CorA family of proteins have been focused on CorAs from *Thermotoga maritima* (TmCorA) and *Methanocoldococcus jannaschii* (MjCorA)^4,6,8,9,12–15^. The initial functional characterization of the family was done with orthologous CorA from *Salmonella typhimurium*^16,17^ which was later extended by the vast amount of data on TmCorA generated by different groups in an attempt to understand its transport mechanism. This led to a situation that only TmCorA is characterized in a great detail by various techniques (including but not limited to transport assays, electrophysiology, Molecular Dynamics simulations, mutagenesis) but for other members such characterization is rather scarce. With the goal to extend such a functional characterization on other members of CorA family we performed the extensive *in vitro* functional characterization of CorA from *M. jannaschii* and of homologous zinc transporter ZntB^18^ in comparison with CorA from *T. maritima*. Our results show that similarly to TmCorA, both MjCorA and EcZntB are not highly selective, and support the hypothesis that CorA and ZntB proteins might utilize different transport mechanisms.

## Results

**Figure 2:**
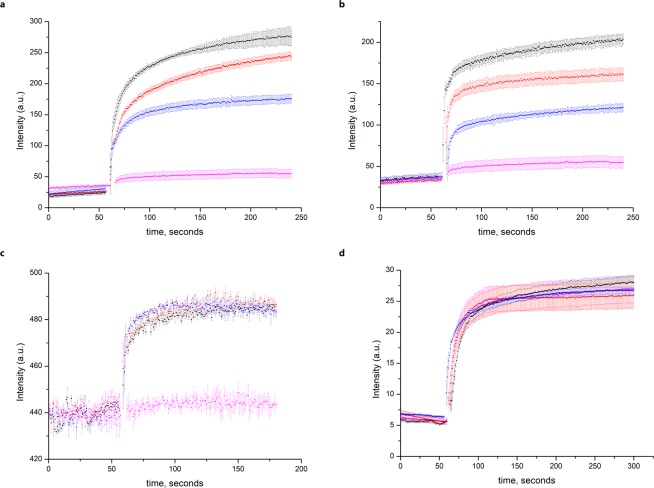
Transport of different cations assayed by the fluorophores trapped inside the proteoliposomes. Dequenching of Fluozin-1 dye fluorescence with (**a**) TmCorA and (**b**) MjCorA (added at 1 min and color-coded: black—25 μM Zn^2+^, red—25 μM Cd^2+^, blue—100 μM Ni^2+^, magenta—empty liposomes with 25 μM Zn^2+^). **c** Dequenching of FluoZin-3 fluorescence during transport of Mg^2+^ via red—ZntB, blue—TmCorA, black—MjCorA, magenta—empty liposomes. 100 μM Mg^2+^ was added at 1 min. (**d**) Incapability of these proteins to transport Al^3+^ as registered by morin dye (the same coloring as in (**c**), 200 μM Al^3+^ was added at 1 min). Error bars represent s.e.m. from three or more technical replicates of independent batches of proteoliposomes.

Purified CorA proteins from *T. maritima* and *M. jannaschii* and ZntB from *E.coli* (Supplementary Fig. 2) were reconstituted into proteoliposomes and their transport activity was assayed as described previously^18^. Both TmCorA and MjCorA readily transported Zn^2+^, Cd^2+^, Co^2+^ and Ni^2+^ similarly to EcZntB as seen in the experiments with the Fluozin-1 dye (Fig. 2a,b); all three proteins readily transport Mg^2+^ as registered with Fluozin-3 dye (Fig. 2c), but are not capable to transport Al^3+^ as seen in the experiments with morin dye (Fig. 2d).

**Figure 3:**
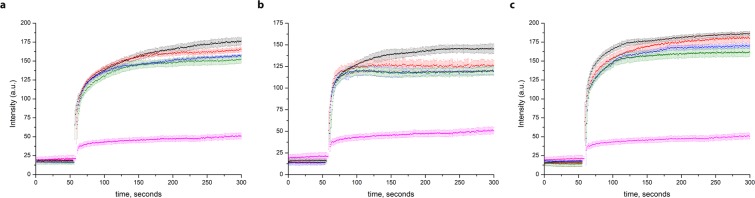
The incapability of hexamminecobalt(III) chloride (CoHex) to inhibit the transport of Zn^2+^ by proteoliposomes with (**a**) TmCorA, (**b**) MjCorA and (**c**) ZntB. Black—25 μM Zn^2+^ with no added CoHex, red— 25 μM Zn^2+^  + 1 μM CoHex, blue—25 μM Zn^2+^  + 10 μM CoHex, green—25 μM Zn^2+^  + 50 μM CoHex, magenta—empty liposomes with 25 μM Zn^2+^  + 50 μM CoHex. All substrates were simultaneously added at 1 min. Higher concentrations of CoHex led to the collapse of proteoliposomes (see Supplementary Fig. 1). Error bars represent s.e.m. from three or more technical replicates of independent batches of proteoliposomes.

Surprisingly the transport activity of TmCorA and MjCorA was not efficiently inhibited by Hexamminecobalt(III) chloride, the known CorA inhibitor^19,20^ (Fig. 3).

**Figure 4:**
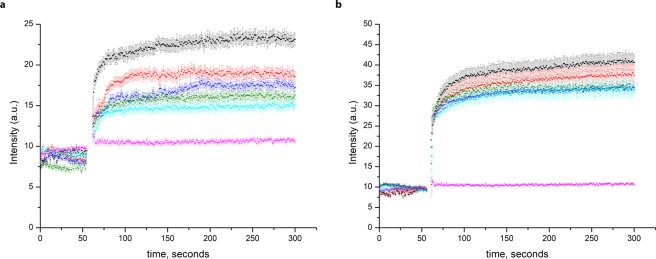
transport competition assays with Co^2+^ and Mg^2+^ in the proteoliposomes, loaded with FluoZin-1, with (**a**) MjCorA and (**b**) TmCorA, (black—100 μM CoSO_4_ with no added MgSO_4_, red—100 μM CoSO_4_ + 100 μM MgSO_4_, blue —100 μM CoSO_4_ + 200 μM MgSO_4_, green—100 μM CoSO_4 + _500 μM MgSO_4_, light blue—100 μM CoSO_4_ + 1000 μM MgSO_4_, magenta—empty liposomes with 100 μM CoSO_4_ + 1000 μM MgSO_4_). All substrates were simultaneously added at 1 min. Error bars represent s.e.m. from three or more technical replicates of independent batches of proteoliposomes.

To test whether either of CorA has a preference for Co^2+^ over Mg^2+^ we performed competition assay experiments. We compared the transport of Co^2+^ by TmCorA and MjCorA in the absence of Mg^2+^ and in the presence of 100–1000 µM of Mg^2+^ (Fig. 4). Whereas transport of Co^2+^ via MjCorA was significantly affected already by addition of 100 µM of Mg^2+^ (Fig. 4a) transport of Co^2+^ via TmCorA was not much affected even in the presence of 1 mM of Mg^2+^ (Fig. 4b). This result supports the previous hypothesis that TmCorA might be a Co^2+^-selective channel^21^.

**Figure 5:**
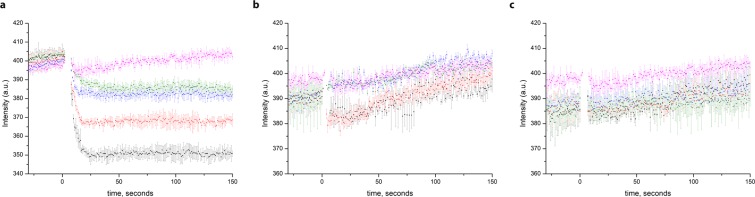
Quenching of the pH-dependent fluorophore ACMA at different Zn^2+^ concentrations in proteoliposomes with (**a**) EcZntB **(b**) TmCorA and (**c**) MjCorA (addition of Zn^2+^ after baseline stabilization, black—50 μM Zn^2+^, red—25 μM Zn^2+^, blue—5 μM Zn^2+^, green—1 μM Zn^2+^, magenta—empty liposomes with 50 μM Zn^2+^). Error bars represent s.e.m. from three or more technical replicates of independent batches of proteoliposomes.

In stark contrast with ZntB, both TmCorA and MjCorA do not seem to transport protons as seen in the experiments with ACMA dye (Fig. 5), supporting our previous hypothesis that ZntB and CorA proteins evolved to use different transport mechanisms despite the same general architecture.

**Figure 6:**
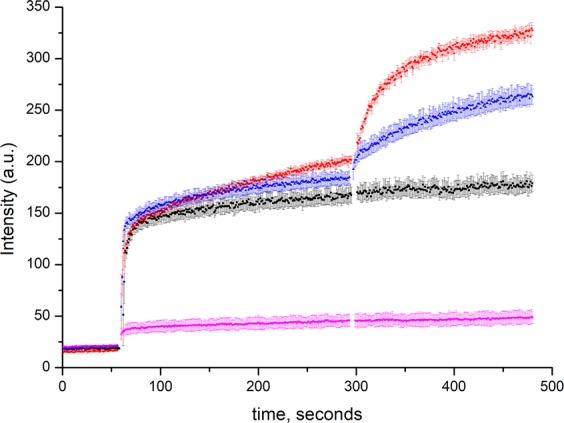
Effect of membrane potential on the transport of Zn^2+^ (added after 1 min) assayed by the fluorophore FluoZin-1 trapped inside the proteoliposomes with EcZntB (black), TmCorA (red) and MjcorA (blue) and empty liposomes (magenta). 1 μM of valinomycin were added after 5 min. Error bars represent s.e.m. from three or more technical replicates of independent batches of proteoliposomes.

This is further corroborated by the fact that ZntB and CorA respond differently to the presence of membrane potential: a creation of membrane potential of −116 mV by addition of valinomycin to proteoliposomes leads to the enhanced transport via CorA but not ZntB proteins (Fig. 6).

**Figure 7:**
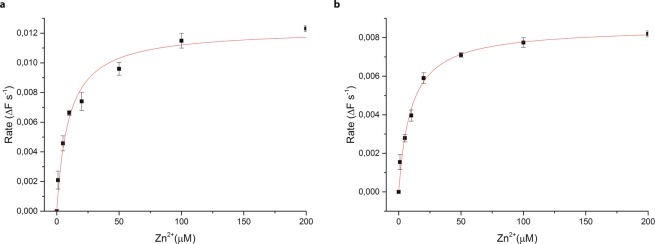
Rate of transport dependence on Zn^2+^ concentration in (**a**) TmCorA **(b)** MjCorA. The solid lines represent the fit to the Michaelis–Menten equation (based on FluoZin-1 experiments). Error bars represent s.e.m. from three or more technical replicates of independent measurements.

The rates of transport, as well as K_m_ values of 9.5 μM and 9.9 μM for TmCorA and MjCorA respectively (Fig. 7), are similar to the previously reported K_m_ value of 7.5 μM for EcZntB^18^.

## Discussion

CorA proteins are the most characterized representatives of 2-TM-GxN family of transporters/channels, which additionally includes ZntB, Alr, Mrs2 and other proteins^22–26^. However there is an obvious knowledge imbalance within CorA subfamily itself, where basically only TmCorA is extensively characterized both functionally^17,27^ and structurally^4,8,9,12,14^. In an attempt to mend it we performed the extensive *in vitro* functional characterization using fluorescence-based transport assays on two other representatives of 2-TM-GxN family, for which the full-length structures are available, namely on archaeal MjCorA^6^ and bacterial EcZntB^18^ in parallel with TmCorA. Using these fluorescence-based transport assays on proteins reconstituted into proteoliposomes we tried to answer whether there is a high substrate specificity within subfamilies – a controversial issue originating from an array of different experiments, including whole cell uptakes, isothermal titration calorimetry and patch clamp experiments and structural models of CorA and ZntB proteins which do not always agree^4,7,18–21,28–30^. Our results show that despite there is a strict selection of divalent over trivalent cations (Fig. 2) for all studied members, the uptake of divalent ions is rather promiscuous. For example, it was assumed that ZntB proteins are strictly selective for Zn^2+^ over Mg^2+^ and *vice versa* for CorA. To monitor the intraliposomal accumulation of Mg^2+^ we used Fluozin-3 dye and not Mag-fura 2 dye as we found the performance of the latter not satisfactory in our experimental setup. The transport of Mg^2+^ via ZntB is comparable with one via TmCorA and MjCorA (Fig. 2c). Similarly, both TmCorA and MjCorA readily transport Zn^2+^ (Fig. 2a,b). Furthermore, for TmCorA it has been shown that in absence of any Mg^2+^ (which is rather a non-physiological) it becomes rather a non-selective divalent channel^30^. Taking into account similarity among studied cations it is feasible to assume that the single main recognition pattern is indeed the hydration radius (close to 2.1 Å) and octahedral arrangement of water molecules in the first hydration shell of a cation as it was proposed earlier^18,31,32^, however that fails to explain the fact why TmCorA prefers Co^2+^ as its substrate even in the presence of 1 mM Mg^2+^ (Fig. 4b). To this moment the most plausible explanation is the presence of additional recognition patterns – such as threonine residues inside the pore^8^, however more systematic study involving more members of both subgroup A (supposedly Co^2+^ -selective channels) and B (supposedly true Mg^2+^ -channels)^33^ is necessary. This result also disfavors the explanation that since TmCorA reside in water habitat it should just prefer Mg^2+^ (ref. ^15^), as the exact elemental pattern in its preferential dwelling (hot springs and hydrothermal vents) might be very different from the normal water composition^34^. If this is correct then basically the preferred substrate will be dictated by the environmental milieu – organisms exposed to high Co^2+^ and low Mg^2+^ evolved to scavenge Co^2+^ more efficiently; the observation that some enzymes of *T. maritima* are cobalt-dependent^35,36^ supports this hypothesis. Furthermore, the results of complementation assay of TmCorA in the salmonella strain devoid of all Mg^2+^ transporters^19^, thermostability assays and competition studies^21^ all indicate that Mg^2+^ is not the most preferred substrate for TmCorA and electrophysiological measurements on TmCorA expressed in oocytes revealed that its affinity for Co^2+^ is ~ 10 times higher than for Mg^2+^ (ref. ^37^).

Another evidence for possible extra selectivity features is the metallotransportosome of *Cupriavidus metallidurans*, which encodes three different CorA proteins (CorA_1_-CorA_3_, with sequence identity below 7%) as well as ZntB protein^7,38^. Interestingly, CmCorA_1_ and CmCorA_2_ are involved in Ni^2+^ import, whereas all three forms can transport Zn^2+^ and CmCorA_123_ heterotrimer is responsible for the import of Co^2+^ (ref. ^7^). Clearly, the structural and functional *in vitro* characterization of *C. metallidurans* transporters will be essential to pinpoint residues responsible for such substrate specificity.

A puzzling observation we made is that hexamminecobalt(III) chloride (CoHex) is not a potent inhibitor (Fig. 3) of CorA and ZntB proteins at least under our experimental conditions. The previously reported IC_50_ values of 0.5–1 µM for StCorA obtained with the radioactive ^63^Ni^2+^ whole cell uptakes^20^ were taken as a reference, however CoHex concentrations up to 50 µM showed little impact on the transport uptake by TmCorA and MjCorA (Fig. 3). We noticed that for the homologous yeast Mrs2 channel^39^ and for TmCorA reconstituted in liposomes^19^ the inhibition of transport was observed with the cobalt hexamine concentration of 1 mM. Furthermore, two-electrode voltage clamp experiments revealed the similar value of K_m_ of 0.9 ± 0.4 mM^30^. We tried performing our uptake experiments in the presence of 0.2–1 mM range of CoHex, however at such high concentrations it led to the collapse of proteoliposomes (Supplementary Fig. 1). We can only speculate why it did not work in our case (different lipid composition, reconstitution ratio, etc.) but it might be as well as in the published work with TmCorA in the presence of 1 mM CoHex the authors observed the collapse of proteoliposomes and not the inhibition effect. Furthermore, in the aforementioned work on Mrs2 complete inhibition was not observed^39^. Interestingly, thermal shift assays studies on MjCorA revealed that CoHex exerts the stabilization effect on the protein (stabilization from 76.7 to ~ 85 °C), albeit it is considerably lower than Co^2+^ ion itself (stabilization up to 95 °C)^40^. Altogether this might indicate that CoHex indeed binds to CorA proteins but either not with the high affinity or not exactly at the selectivity filter.

Our results also provide further evidence that CorA and ZntB proteins diverged to use different transport mechanisms. First of all, CorA proteins seem not to utilize any proton gradient in contrast with ZntB (Fig. 5). In EcZntB proteoliposomes under conditions of equal pH inside and outside, 9-amino-6-chloro-2-methoxyacridine (ACMA) dye senses the buildup of pH gradient upon Zn^2+^ transport and the fluorescence is quenched (Fig. 5a). In case of TmCorA and MjCorA apparently there is no co-transport of H^+^ thus the fluorescence is more or less at the same level (Fig. 5b,c).

In line with the previous reports^19,41^ the influx of Mg^2+^ via CorA proteins is driven by the membrane potential (Fig. 6). Addition of valinomycin to CorA proteoliposomes loaded with 25 mM potassium chloride, leads to the fast escape of potassium ions and build-up of membrane potential to the −116 mV enhancing the transport of divalent cations. The similar behavior of TmCorA was shown before in the experiments with Mag-fura 2 dye^19^, however the enhancement of transport was less pronounced. This discrepancy could be caused by difference in the experimental setup and liposome preparation and / or fluorescent properties of different dyes. In the yeast mitochondria expressing Mrs2 (in KCl buffer), the influx of Mg^2+^ was significantly reduced upon addition of valinomycin, which dissipated mitochondrial membrane potential^39^.

The emerging picture is that in the 2-TM-GxN family the homo- pentameric fold evolved for recognition of similar divalent cations - such as Mg^2+^, Co^2+^, Ni^2+^, Zn^2+^. However, in the particular milieus, where a certain cation is prevailing, specificity might have evolved. Furthermore, for not yet discovered reasons, some members, such as CorA and Mrs2 evolved to be highly-conductive magnesium channels^24,30^, whereas others such as ZntB and Alr proteins became proton-coupled sympoters^18,22^. Clearly there are still open questions, such as what is the actual mode of CoHex binding to CorA proteins, and how some members can be involved in the transport of divergent Al^3+^ cation (1.9 Å first hydration shell radius vs ~ 2.1 Å for aforementioned cations). The elucidation of structures as well as thorough functional characterization of other members of 2-TM-GxN is necessary to answer such questions and to fully understand the transport of ions in this family of proteins.

## Methods

### Cloning

TmCorA and MjCorA were cloned into pNIC28-Bsa4 vector encoding an N-terminal 6xHis-tag and a tobacco etch virus protease cleavage site. The full-length CorA genes were amplified from genomic DNAs of *Thermotoga maritima* and *Methanocaldococcus jannaschii* (DSMZ, Germany). The expression vector was constructed using ligation independent cloning with primers for TmCorA (forward 5′-TACTTCCAATCCATGGAGGAAAAGAGGCTGTCTGC-3′ and reverse 5′-TATCCACCTTTACTGTCACAGCCACTTCTTTTTCTTG-3′) and MjCorA (forward 5′-TACTTCCAATCCATGATTACGGTAATTGCTATAGC-3′ and reverse 5′-TATCCACCTTTACTGCTAAATCCATCCTGACCTTC-3′).

### Protein expression and membrane vesicle preparation

TmCorA, MjCorA and EcZntB proteins were expressed in the same way according to the previously established protocol^18^: expression of target protein was performed in a 5-l flask containing 2 l of LB medium (10 g l^−1^ Bacto trypton, 5 g l^−1^ Bacto yeast extract, 10 g l^−1^ NaCl), supplemented with 50 ug ml^−1^ kanamycin and 34 ug ml^−1^ chloramphenicol. The *E. coli* BL-21(DE3) cells with the needed plasmid were grown at 37 °C, 200 rpm to an OD_600_ of 0.8, with an induction by addition of 0.1 mM IPTG. After 3 h of expression the cells were collected by centrifugation (15 min, 7,446 g, 4 °C), washed in buffer A (50 mM Tris/HCl, pH 8.0) and resuspended in the buffer B (50 mM Tris/HCl, pH 8.0, 250 mM NaCl, 10% glycerol). Membrane vesicles were prepared as described previously^18^ and were either prepared immediately, or the resuspended cells were stored at −80 °C after flash freezing in liquid nitrogen. Before membrane vesicle preparation, 1 mM MgSO4 and 50–100 ug ml^−1^ DNase were added to the cells. The cells were lysed by high-pressure disruption (Constant Cell Disruption System Ltd, UK, two passages at 25 kPsi for *E. coli* cells, 5 °C) and cell debris was removed by low-speed centrifugation (30 min, 12,074 g, 4 °C). Membrane vesicles were collected by ultracentrifugation (120 min, 193,727 g, 4 °C), and resuspended in buffer C (50 mM Tris/HCl, pH 8.0, 150 mM NaCl, 15% glycerol) to a final volume of 5 ml per 1 l of cell culture. Subsequently, the membrane vesicles were aliquoted, flash frozen in liquid nitrogen and stored at −80 °C.

### Protein purification

Protein purification was done as described previously^18^. Membrane vesicles were thawed rapidly and solubilized in buffer D (50 mM Tris/HCl, pH 8.0, 150 mM NaCl, 10 mM imidazole, 10% glycerol, 1% (w/v) n-dodecyl-β-D-maltopyranoside (DDM, Anatrace)) for 1 h at 4 °C, while gently rocking. Unsolubilized material was removed by centrifugation (30 min, 442,907 g, 4 °C). The supernatant was incubated for 1 h at 4 °C under gently rocking with Ni^2+^-sepharose resin (column volume of 0.5 ml), which had been equilibrated with 10 CV of buffer E (50 mM Tris/HCl, pH 8.0, 250 mM NaCl, 50 mM imidazole, 0.03% DDM). Subsequently, the suspension was poured into a 10-ml disposable column (Bio-Rad) and the flow through was collected. The column material was washed with 10 ml of buffer E. The target protein was eluted in three fractions of buffer F (50 mM Tris/HCl, pH 8.0, 250 mM NaCl, 500 mM imidazole, 0.03% (w/v) DDM) of 200, 750 and 500 µl, respectively. 2 mM of EDTA was added to the second elution fraction to remove co-eluted Ni^2+^ ions and any residual divalent cations. Subsequently, the second elution fraction was purified by size-exclusion chromatography using a Superdex 200 10/300 gel filtration column (GE-Healthcare), equilibrated with buffer G (50 mM Tris/HCl, pH 8.0, 250 mM NaCl, 0.03% (w/v) DDM). After size-exclusion chromatography, the fractions containing the target protein were combined and used directly for proteoliposome reconstitution.

### Reconstitution into proteoliposomes

Reconstitution in proteoliposomes was performed as described previously^18^: polar lipids of *E. coli* and egg phosphatidylcholine (in 3:1 (w/w) ratio) were dissolved in chloroform, then dried in a rotary evaporator and subsequently resuspended in buffer containing 50 mM KPi, pH 7.5 to the concentration of 20 mg ml^−1^. After three freeze-thaw cycles, large unilamellar vesicles (LUVs) were obtained and stored in liquid nitrogen. To prepare proteoliposomes, LUVs were extruded through a 400-nm-diameter polycarbonate filter (Avestin, 11 passages). Obtained liposomes were diluted to 4 mg ml^−1^ in buffer H (50 mM HEPES, pH 7.5) or buffer I (50 mM HEPES, pH 6.5) and subsequently destabilized beyond R_sat_ with Triton X-100. The target purified protein was added to the liposomes at a weight ratio of 1:250 (protein/lipid), followed by detergent removal using Bio-beads (50 mg ml^−1^, four times after 0.5 h, 1 h, 2 h and overnight incubation). Afterwards, proteoliposomes were collected by centrifugation (25 min, 285,775 g, 4 °C) and resuspended in buffer H or buffer I to a lipid concentration of 10 mg ml^−1^. Finally, after three freeze-thaw cycles, obtained proteoliposomes were stored in liquid nitrogen until subsequent experiments.

### Fluorescent transport assays

Transport of metals was measured according to the previously established protocol^18^. Zinc transport was measured with the Zn^2+^-sensitive fluorophore FluoZin-1 (ThermoFisher, USA). To avoid bleaching of the fluorophore, the sample was shielded from the direct light as much as possible. FluoZin-1 (stock concentration 3 mM in H_2_O) was added to a final concentration of 5 μM to the proteoliposomes. FluoZin-1 encapsulation was performed by three freeze-thaw cycles and subsequent extrusion through 0.4 µm polycarbonate filters. Extravesicular dye was removed from approximately 500 μl of liposome suspension by size exclusion chromatography on a 2 ml Sephadex G-75 column equilibrated with buffer H or I. Proteoliposomes were collected by ultracentrifugation (25 min, 285,775 g, 4 °C), and the supernatant was removed. Proteoliposomes were resuspended with 10 μl buffer H or I per 2.5 mg of proteoliposomes (protein to lipid ratio 1:250). Transport assays were initiated by the addition of 10 mM stock solution of zinc acetate to the desired final concentration. For each measurement, 0.3 mg of proteoliposomes was diluted in 1 ml of desired buffer. A fluorescence time course was measured in a 1-ml cuvette with a stirrer (350 rpm) using an excitation wavelength of 490 nm and an emission wavelength of 525 nm. Experiments with empty liposomes were performed in parallel as controls. Initial transport rates (ΔF s^−1^) were calculated by performing a linear regression on the transport data between 1 and 10 s after addition of zinc acetate. The resulting data was fitted to a Michaelis-Menten equation. All measurements were at least triplicated.

To investigate the inhibition effect of hexamminecobalt (III) chloride (CoHex), the proteoliposomes loaded with FluoZin-1 were preincubated with various concentrations of CoHex from 1 μM to 1 mM for 3 minutes, after that 25 μM zinc acetate was added. All other steps were performed in the similar way as described above. Experiments with empty liposomes were performed in parallel as controls. All measurements were triplicated. To check the ability of target proteins to transport Al^3+^, the proteoliposomes were prepared the same way as for FluoZin-1, but instead loaded with 3 μM morin (Sigma-Aldrich). A fluorescence time course was measured in a 1-ml cuvette with a stirrer using an excitation wavelength of 420 nm and an emission wavelength of 500 nm; 50 μM AlCl_3_ was added after 1 minute of equilibration time. Experiments with empty liposomes were performed in parallel as controls. All measurements were triplicated. Magnesium transport was measured by FluoZin-3 (ThermoFisher, USA). All preparations of the proteoliposomes were the same as with FluoZin-1 except FluoZin-3 (stock concentration 1 mM in H_2_O) was added to a final concentration of 5 μM to the proteoliposomes. A fluorescence time course was measured in a 1-ml cuvette with a stirrer using an excitation wavelength of 494 nm and an emission wavelength of 516 nm. After 3 minute of the baseline’s stabilisation 100 μM MgSO_4_ was added. Experiments with empty liposomes were performed in parallel as controls. All measurements were triplicated. H^+^ transport assays were performed as described previously^18^: the lumenal buffer of the proteoliposomes was exchanged for buffer J (5 mM HEPES pH 6.7) by resuspension of the liposomes in this buffer followed by three freeze-thaw cycles and extrusion through 0.4 μm polycarbonate filters. Proteoliposomes were collected by ultracentrifugation (25 min, 285,775 g, 4 °C), and the supernatant was removed. Proteoliposomes were resuspended with 10 μl buffer J per 2.5 mg of proteoliposomes (protein to lipid ratio 1:250). For each measurement, 0.3 mg of proteoliposomes was diluted in 1 ml of buffer K (5 mM HEPES, pH 6.7, 150 nM ACMA). A fluorescence time course was measured in a 1-ml cuvette with a stirrer using an excitation wavelength of 419 nm and an emission wavelength of 483 nm; zinc acetate was added after 3 minutes of equilibration time. Experiments with empty liposomes were performed in parallel as controls. All measurements were triplicated.

### Data analysis

The structural figures were produced with an open source version of Pymol (https://github.com/schrodinger/pymol-open-source). The sequence alignment was produced with T-coffee^42^ (http://tcoffee.crg.cat/apps/tcoffee/index.html) and annotated with Espript 3.0 (ref. ^43^) (http://espript.ibcp.fr). The statistical analysis was performed in Excel (Microsoft Corp.) and the final graphs were produced in Origin Pro 7 (OriginLab Corp.)

## Supplementary information

Supplementary Information.

## Data Availability

All data reported in this research are available from the corresponding author on reasonable request.
