# Supplementary material for: Cation permeability in CorA family of proteins
Source: Sci Rep. 2020 Jan 21;10:840. doi: 10.1038/s41598-020-57869-z (PMC6972854; doi:10.1038/s41598-020-57869-z)
Supplement: Supplementary file 1 — Supplementary Information. [file 41598_2020_57869_MOESM1_ESM.pdf]

Cation permeability in CorA family of proteins

Artem Stetsenko, Albert Guskov

Supplementary Figure 1

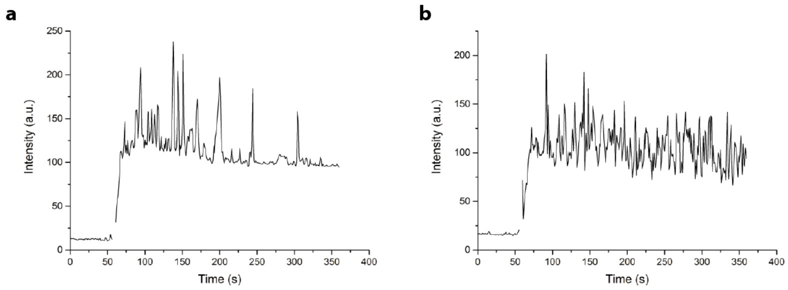

Collapse of proteoliposomes with (**a**) TmCorA (**b**) MjCorA under addition of > 200 µM Hexamminecobalt(III) chloride as seen from the instability of the fluorescence time course.

Supplementary Figure 2

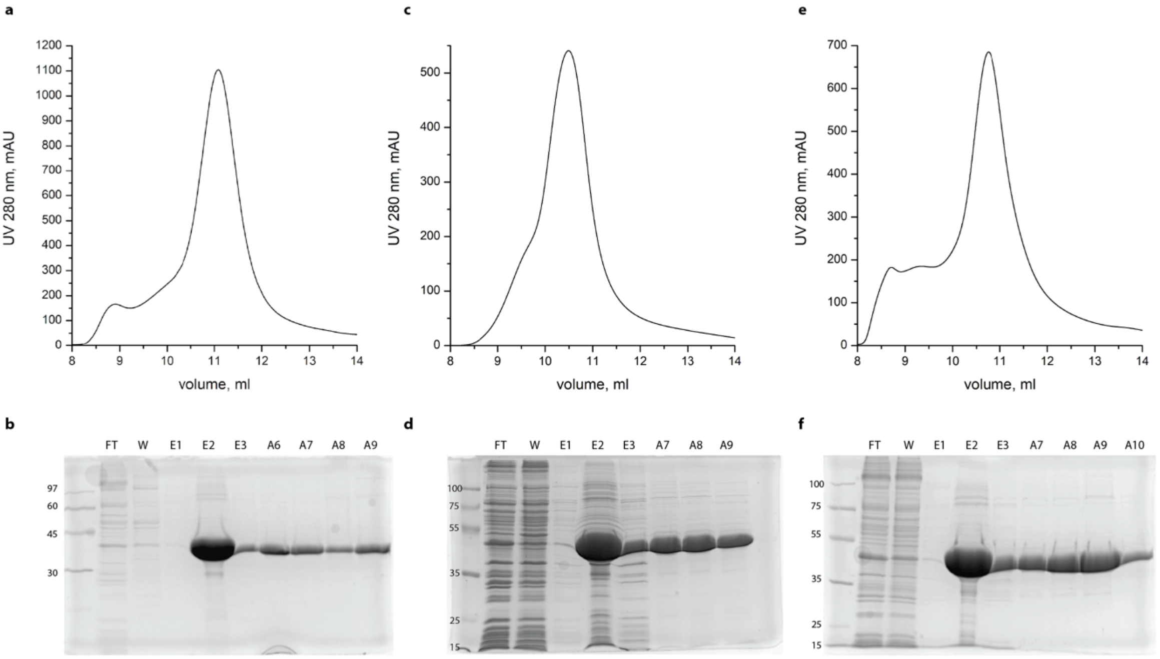

Protein purification. Size-exclusion chromatography profiles and the corresponding SDS-PAGE gels of (**a-b**) EcZntB, (**c-d**) TmCorA and (**e-f**) MjCorA. Please note different markers in SDS-PAGE gels were used for ZntB and CorA proteins. Only fraction A8 was used for experiments.
